# Neurophysiological Markers of Internet Gaming Disorder: A Literature Review of Electroencephalography Studies

**DOI:** 10.7759/cureus.17866

**Published:** 2021-09-10

**Authors:** Saima Kashif, Samiksha Pandey, Zain I Warriach

**Affiliations:** 1 Department of Research, California Institute of Behavioral Neurosciences & Psychology, Fairfield, USA; 2 Department of Research, Division of Clinical & Translational Research/Larkin Health System, Miami, USA

**Keywords:** electroencephalography (eeg), internet gaming disorder (igd), biomarkers, event-related potential (erp), treatment, gaming disorder(gd)

## Abstract

Pathological online gaming is a relatively newer psychiatric disorder. It is the second behavioral addiction (after internet gambling disorder) included in the Diagnostic and Statistical Manual of Psychiatric Disorders (DSM), 5^th^ edition as “Internet Gaming Disorder” (IGD). Recent research is showing high morbidity associated with IGD, thus encouraging researchers to find valid and reliable biomarkers of IGD. So that, early diagnosis and proper treatment could limit the high disability level accompanying excessive online gaming. As electroencephalography (EEG) is a non-invasive and relatively easily available diagnostic technique, we aimed at collecting EEG studies that investigated EEG changes associated with IGD, with a specific focus on finding diagnostic and predictive neurophysiological biomarkers of IGD. We searched PubMed and Google Scholar for EEG studies of IGD. We eliminated those EEG studies that were not focused on finding biomarkers. Scale for the Assessment of Narrative Review Articles (SANRA) is followed in the writing of this review article. Our results showed that increased slow-wave resting-state activity and reduced P300 and N100 can serve as useful IGD diagnostic markers of IGD. Moreover, increased resting-state theta activity can be a predictive biomarker of IGD. Lastly, increased late low potentials (LLPs) can be specific trait markers of cue-induced cravings in IGD.

## Introduction and background

In the modern world of digitalization, uncontrolled online gaming emerged as a global problem not only among children and adolescents but also among adults. In 2013, American Psychiatrist Association (APA) included problematic gaming in the third section of the Diagnostic and Statistical Manual (DSM-V) as “Internet Gaming Disorder” (IGD). Initially, in DSM-V, IGD was taken as a condition that needed further research before being added to the main manuscript [1]. Shortly after APA, excessive online gaming got included in the 11th edition of the International Classification of Diseases (ICD-11) as “Pathological Gaming” (PG), thus accepting abnormal gaming behavior as a psychiatric condition that requires proper diagnosis and management [2]. Diagnostic criteria of IGD, in DSM-V, are mainly derived from substance use disorder and pathological gambling disorder criteria. It consists of nine core symptoms including, tolerance, preoccupation, withdrawal, failure to reduce or stop gaming regardless of a desire to do so, abandoning other activities for gaming, continuing gaming despite substantial problems, cheating to cover up the extent of gaming, using gaming as a distraction, and jeopardizing or losing future opportunities (e.g., educational, career) or relationships due to uncontrolled gaming [1,2].

Remarkably, IGD prevalence ranges from 0.8% to 11.8% in European populations to above 10% in some Asian countries, affecting people regardless of age, ethnicity, educational level, or geographical distribution [3]. However, in some technologically advanced countries, online gaming addiction is more prevalent. Many studies confirmed the association of IGD with multiple psychosocial issues including, sleep difficulties, poor school performance, oppositional behavior, and attention deficit hyperactivity disorder (ADHD) in children [4]. Sometimes these comorbidities could be quite serious like depression, substance addiction, and suicidal behaviors among adolescents and adults [5,6]. In extreme cases, there are also media reports of gaming-induced seizures and deaths [7].

Researchers have identified certain risk factors associated with a high risk of gaming addiction. However, due to the increasing prevalence and associated psychiatric and social problems, researchers are now looking for measurable biological indicators or commonly called biomarkers for IGD that can help us in the early diagnosis and treatment of this rapidly rising behavioral condition. In a broad definition, a biomarker is any substance, structure, or process in a body that can be measured objectively [8]. As compared to symptoms of an illness that are subjective, a biomarker is an objective, accurate, and reproducible parameter that can be anything from respiratory/heart rate to neurotransmitter levels in the brain. In addition to their diagnostic importance, predictive biomarkers can also help in measuring treatment response. Despite the fact that most of the research on IGD biomarkers is focused on neuroimaging, neurophysiological techniques could provide more economical, non-invasive, and easily measurable biomarkers to predict disease incidence, severity, prognosis, and response to treatment. Thus, we aimed at collecting electroencephalography (EEG) studies that aimed at finding potential neurophysiological biomarkers of IGD, as EEG is the most useful tool to study neurophysiological markers of any disease or disorder.

EEG is the recording of electrical brain activity through metallic electrodes attached to the scalp in a standard fashion. These recordings taken in absence of any stimulus or activity, when one is relaxed with closed eyes are called “resting-state EEG.” These recordings are classified into four types: alpha; beta; delta; and theta, according to frequency (Hz or cycles/second) and amplitude. Resting-state EEG is commonly used in research to find neurophysiological biomarkers, as it is an efficient and non-invasive technique to explore spontaneous neural activity. Furthermore, EEG scans recorded in a specific time frame after the presentation of a certain stimulus (for example, auditory or visual) to the person, are called event-related potentials (ERP). These waves are then analyzed according to polarity (positive or negative) and latency after stimulus presentation (for example, in milliseconds). Thus, ERP is an efficient research tool to study neural activities associated with cognitive and attentional deficits in certain conditions. EEG coherence analyses can be recorded when an individual is in a resting state or during a brain activity (presentation of an auditory or visual deficit). It is an analysis of coordination between activities of different brain areas. Late low potential (LLP) is a type of ERP, in which midline brain recordings are taken during emotionally salient stimuli. It has been used extensively in the past for investigating attentional bias in various types of addictions [9].

We hope that this article will highlight some important key facts associated with IGD biomarkers and will reveal new dimensions for future research in this respect.

## Review

Materials and method

PubMed and Google Scholar databases were systematically searched for relevant publications to identify studies that investigated EEG findings in IGD individuals. In addition, we also included researches that compared EEG recordings of IGD subjects to other addictions. Keywords used were Internet Gaming Disorder, Pathological Gaming, biomarkers, quantitative Electroencephalography, Event-Related Potential, and treatment. Initially, 21 studies were found that studied EEG recordings in IGD. Abstracts of relevant publications were further evaluated for applicability to the idea of potential biomarkers of IGD. In the end, we were left with nine studies that found or suggested neurophysiological biomarkers of IGD. No restrictions were applied on language or time of publication.

Discussion

We identified nine studies relevant to our topic. Out of these nine studies, five used EEG, three used ERP, and one investigated LLP in IGD.

Kim et al. studied EEG recordings of IGD individuals in an attempt to find diagnostic and prognostic biomarkers of IGD. Resting-state recordings of IGD and normal healthy controls (HCs) were compared, before and after six months of treatment with selective serotonin reuptake inhibitors (SSRIs). A comparison was made between pre-treatment recordings of the IGD group and the control group and between pre-treatment and post-treatment EEG recordings of the IGD group. Results showed increased slow-wave activity (both beta and theta band) at baseline in the IGD group. However, after six months of treatment with SSRIs, this heightened slow-wave activity and addiction symptoms got normalized. In addition, higher theta band activity was found to be associated with better control of addiction symptoms in post-treatment recordings. The study concluded that increased slow-wave activity can be considered a valuable IGD neurophysiological diagnostic marker, but increased theta activity specifically can serve as a predictive marker [10].

Similar findings were confirmed in another study by Choi, et al. They compared EEG scans of IGD subjects having a comorbid psychiatric illness (depression or anxiety disorder) and HCs. IGD subjects (with comorbidity) were found to have increased resting beta and theta activities at baseline. Moreover, increased theta activity along with symptoms of gaming addiction showed significant improvement after six months of treatment. Thus, suggesting increased slow-wave activity as a diagnostic marker, while increased theta activity as a predictive marker of IGD [11].

Park et al. compared resting-state EEG coherence analyses before and after six months of treatment with SSRIs in IGD subjects. Bilaterally increased gamma and beta intrahemispheric coherence along with increased delta intrahemispheric coherence of the right hemisphere at baseline was recorded. Moreover, this increased coherence did not show any significant improvement, despite improvement in IGD symptoms after six months of pharmacotherapy. It was concluded that increased intrahemispheric fast-frequency coherence can be a useful diagnostic marker of IGD. However, it showed no significance in predicting treatment response [12].

Son et al. compared resting-state EEG patterns of IGD, alcohol use disorder (AUD), and healthy subjects in an attempt to find neurophysiological markers unique to IGD. Results showed weaker absolute beta power in IGD subjects compared to AUD and HC groups. Thus, it was suggested that lower absolute beta power can be a potential diagnostic marker of IGD [13].

Despite having increased slow-wave activity in the resting state, IGD subjects exhibited decreased theta, alpha, and beta band activities in the left frontal area during their favorite gameplay, in another study. In addition, a strong negative correlation was found between left theta power and symptom severity in IGD subjects. The study concluded that left theta power specifically can be a strong marker for dysfunctional cognitive control in IGD individuals [14].

We found three studies investigating ERPs, and one study investigating LLPs in IGD individuals.

Park et al. investigated alterations in P300 amplitude in IGD individuals during an auditory oddball task. IGD subjects, compared to HCs, exhibited reduced P300 amplitude in the midline centro-parietal regions in response to deviant tones. In addition, a negative correlation between decreased P300 and IGD symptom severity was found. Thus, suggesting “reduced P300” as a candidate biomarker for IGD, which can also explain the neurophysiological mechanism underlying impaired auditory processing in IGD subjects [15].

Park et al., in peruse of finding predictive neurophysiological markers of IGD, compared ERP scans before and after six months of outpatient treatment with SSRIs. No significant improvement in reduced P300 both in responders and non-responders to treatment was found, thus concluding that reduced P300 has no predictive value [16]. Park et al. in another study, compared alterations in both P300 and N100 amplitudes using an auditory oddball task in individuals with IGD, AUD, and HCs. IGD subjects had decreased P300 amplitude in centro-parietal regions, while reduced N100 amplitude in the midline frontal area compared to HCs. As reduced P300 amplitude among IGD and AUD groups was comparable, a study concluded that “reduced N100” could be a valuable specific biomarker of IGD [17].

As attentional bias and cue-induced cravings are chief characteristics of both substance and behavioral addictions, Kim et al. aimed to find biomarkers of attentional bias to gaming cues in IGD, using LLPs. LLP recordings were taken in response to gaming obsessive control disorder (OCD), and neutral cues in IGD, OCD, and healthy subjects. Both IGD and OCD subjects showed heightened LLPs in response to gaming and OCD cues. The study concluded that increased LLPs can serve as a trait neurophysiological marker of cue-induced cravings in IGD [18].

Our review findings (Table 1) are in line with findings of previous reviews about neurophysiological findings in IGD [19].

**Table 1:** Summary of findings EEG: electroencephalography; IGD: internet gaming disorder; AUD: alcohol use disorder; HC: healthy controls, ERP: event-related potential; LLP: late low potentials; OCD: obsessive-compulsive disorder

Reference	EEG technique used	Control groups	Findings
Kim et al. [10]	Resting-state EEG	Age-matched healthy controls	Increased slow wave activity can be considered a biomarker of IGD, increased theta activity can serve as a predictive biomarker of IGD
Choi et al. [11]	Resting-state EEG	IGD subjects with comorbid psychiatric disorders and HCs	Increased slow wave activity can be considered a diagnostic marker of IGD, increased theta activity can serve as a predictive biomarker of IGD
Park et al [12]	Resting-state EEG coherence analyses	IGD subjects before and after treatment	Increased intrahemispheric fast-frequency coherence can be a useful trait marker for IGD but it has no significance as a predictive marker
Son et al [13]	Resting-state EEG	AUD subjects and HCs	Lower absolute beta power can be used as a diagnostic marker of IGD
Kim et al. [14]	EEG during gaming	HCs	Decreased theta, alpha, and beta band activities in the left frontal area during gaming-diagnostic marker decreased left theta power during gameplay, specifically can be a marker for dysfunctional cognitive control in IGD subjects
Park et al. [15]	ERP	HCs	Reduced P300 amplitude in IGD can serve as a diagnostic marker
Park et al. [16]	ERP	IGD subjects before treatment and after treatment	Reduced P300 in IGD has no value as a predictive biomarker
Park et al. [17]	ERP	AUD subjects, HCs	Reduced P300 amplitude in both IGD and AUD subjects, reduced N100 could be a valuable specific biomarker of IGD
Kim et al. [18]	LLPs	HCs, OCD	Increased LLPs can serve as a trait neurophysiological marker of cue-induced cravings in IGD

It’s worth mentioning here that we also found four other studies that utilized EEG for the investigation of neurophysiological mechanisms in IGD individuals. However, they have no specific emphasis on finding neurophysiological biomarkers of IGD.

Littel et al. aimed at investigating neurobiological mechanisms underlying poor response inhibition and error processing applying go-no-go tasks. Study findings revealed reduced event-related negativity (ERN) amplitudes in response to incorrect trials in excessive gamers compared to casual gamers, correlated to behavioral and self-reported high impulsivity and poor response inhibition among excessive gamers [20]. Duven et al. compared ERP recordings of IGD and HC groups during active gaming in peruse of finding tolerance effects of online gaming. Results showed attenuated P300 in IGD subjects compared to controls in response to rewards. In addition, IGD individuals also had prolonged latency and increased amplitude of N100. Overall, it was concluded that online gaming, similar to other addictions, can cause tolerance [21].

As IGD has higher comorbidity with major depressive disorder (MDD), Youh et al. compared inter-and intrahemispheric electroencephalographic coherence between MDD only and MDD with comorbid IGD individuals, utilizing quantitative EEG. Results showed significantly increased frontal interhemispheric coherence in MDD individuals compared to MDD with the IGD group, which possibly can explain the higher incidence of attentional problems in MDD+IGD subjects. Furthermore, the MDD+IGD group exhibited increased coherence in fronto-temporo-parieto-occipital brain areas, which is speculated as an effect of excessive online gaming [22]. As it is believed that IGD individuals have problems in socializing, and facial expression recognition is the key to good social interaction, Peng et al. investigated facial expression processing among IGD subjects utilizing ERPs. IGD subjects and HCs were presented happy-neutral-sad facial expressions subliminally while participating in the backward masking task. Results revealed lower N170 (a measure of facial expression processing) amplitude in response to neutral expressions compared to happy expressions in happy-neutral context among IGD subjects compared to controls. However, both IGD and control groups had comparable N170 amplitudes in response to sad and neutral expression in a sad-neutral context. These results show deficit facial processing in IGD individuals that might be due to their higher expectations of happy expressions [23].

## Conclusions

On the basis of the present research literature available, increased resting-state slow-wave activity (beta and theta) can be suggested as a valuable neurophysiological biomarker of IGD. In addition, increased resting-state theta activity can also be helpful as a predictive biomarker. Low absolute beta power and increased intrahemispheric fast-frequency coherence are two other diagnostic neurophysiological biomarkers of IGD identified in recent research. Furthermore, raised left frontal theta power during gameplay is a suggested biomarker for impaired cognitive control in IGD individuals.

Regarding ERP, reduced P300 and N100 are two other suggested diagnostic markers of IGD. But they have no correlation with the severity of addiction or improvement in symptoms. Moreover, reduced P300 can be a useful marker of dysfunctional cognitive control in IGD subjects. High LLPs in response to gaming cues can also serve as a neurophysiological biomarker of IGD. Thus, it can be helpful in the identification of individuals with a high risk of relapse.

Despite our best efforts, this study has a number of limitations. First, researches specifically studying IGD biomarkers are extremely limited and there is a need to conduct more research in this respect to find practically applicable biomarkers. Second, there was a lack of standardization as different studies used different criteria and scales to diagnose IGD. Moreover, different studies used different comparison groups like IGD vs. AUD or IGD vs. HCs, etc. Third, in most of the studies conducted on IGD subjects, participants were young males, ignoring the fact that neurophysiological correlates of gaming might differ between males/females and different age groups.
